# Findings, phenotypes, and outcomes in Freeman-Sheldon and Sheldon-Hall syndromes and distal arthrogryposis types 1 and 3: protocol for systematic review and patient-level data meta-analysis

**DOI:** 10.1186/s13643-017-0444-4

**Published:** 2017-03-06

**Authors:** Mikaela I. Poling, José Andrés Morales Corado, Robert L. Chamberlain

**Affiliations:** Guideline Task Force, Department of Applied Physiology, FSRG deGruyter-McKusick Institute of Health Sciences, 6670 Old Elkins Road, Buckhannon, WV 26201 USA

**Keywords:** Freeman-Sheldon syndrome, Craniocarpotarsal dystrophy, Whistling face syndrome, Sheldon-Hall syndrome, Distal arthrogryposis type 1, Distal arthrogryposis type 3, Systematic review, Meta-analysis, Data analysis, Review of reported cases

## Abstract

**Background:**

Freeman-Sheldon and Sheldon-Hall syndromes (FSS and SHS) and distal arthrogryposis types 1 and 3 (DA1 and DA3) are rare, often confused, congenital syndromes. Few studies exist. With reported diagnosis unreliable, it would be scientifically inappropriate to consider articles describing FSS, SHS, DA1, or DA3, unless diagnoses were independently verified, rendering conventional systematic review and meta-analysis methodology inappropriate and necessitating patient-level data analysis (PROSPERO: CRD42015024740).

**Methods/design:**

As part of a clinical practise guideline development process, we evaluate (1) diagnostic accuracy from 1938–2017, using the Stevenson criteria; (2) the most common physical findings, possible frequency clusters, and complications of physical findings amongst patients with FSS; and (3) treatment types and outcomes. All papers reporting diagnosis of FSS, SHS, DA1, and DA3 are included in searching PubMed and Google Scholar from December 2014 to July 2015 and again before final analyses. Patients with FSS are divided into four phenotype-defined sub-types; all patients are grouped by published diagnosis and medical speciality. Significance of physical findings and historical data is evaluated by chi-square. Associations of physical findings and history with diagnosis and treatment outcome are evaluated by Pearson correlation and linear regression analysis. Two-tailed alpha level of 0.05 is used throughout.

**Discussion:**

The need for detailed patient-level data extraction may limit the types of articles included and questions able to be answered. For treatment and psychosocial health outcomes, we anticipate enhanced difficulties, which may limit significance, power, and results’ usability. We hope to outline knowledge gaps and prioritise areas for clinical investigation.

**Systematic review registration number:**

CRD42015024740

Universal Trial Number: U1111-1172-4670

**Electronic supplementary material:**

The online version of this article (doi:10.1186/s13643-017-0444-4) contains supplementary material, which is available to authorized users.

## Introduction

First described by Freeman and Sheldon [1], Freeman-Sheldon and Sheldon-Hall syndromes (FSS and SHS) are highly variable, rare, phenotypically similar but genotypically distinct, and often confused congenital myopathies, despite previous attempts to simplify diagnosis [2]. While Antley et al. (1970) completed a systematic review and statistical analysis of patient-level clinical information from published reports, allelic variation data were not available, and few cases were available for analysis [2]. Stevenson et al. (2006) executed a cohort study of geneticist-referred patients with classic FSS and SHS [3], which limited their cohort, but they were able to define meaningful and strict clinical diagnostic criteria for FSS and SHS. For FSS, they required the following: microstomia, whistling-face appearance (pursed lips), H or V-shaped chin dimpling, prominent nasolabial folds, and major contractures in two or more body regions [3]. For SHS, they required the following: small mouth (not microstomia), prominent nasolabial folds, small but prominent chin, neck webbing, and major contractures in two or more body regions [3].

In FSS, spinal deformities, metabolic and gastroenterological problems, other dysmorphic craniofacial characteristics, and visual and auditory impairments are frequent findings [2, 3]. Some individuals present with minimal malformation; rarely, patients die during infancy as a result of severe central nervous system involvement or respiratory complications. While patients with classic FSS have normal intelligence, a genotypically distinct lethal variant characterised by profound and progressive neurological motor and cognitive impairment exists [4]. For FSS and SHS, the most familial cases are by autosomal dominant inheritance, but expression is often from new allelic variation [3]. Frequency of it is unknown, mostly due to diagnostic uncertainty; there appears to be neither sex nor ethnic preference. Environmental and parental factors are not implicated in pathogenesis.

No systematic review has been completed since the genotype-verified clinical diagnostic criteria [5, 6] were published. Unfortunately, few studies involving either or both FSS and SHS exist, and with reported diagnosis unreliable, it would be scientifically inappropriate to consider articles describing FSS or SHS, unless diagnoses were independently verified. Thus, a conventional systematic review and meta-analysis methodology was inappropriate in this instance, and rigorous patient-level clinical information extraction and aggregation was required.

The systematic review and patient-level data meta-analysis objectives are as follows: to (1) determine diagnostic accuracy from 1938–2017, using the Stevenson criteria [3]; (2) define the most common physical findings, possible frequency clusters and complications of physical findings amongst patients with FSS; and (3) document treatment types and outcomes. Additionally, a myriad of phenotypes are described as FSS—further complicating literature searches and clinical practise guideline development. To evaluate possible differences with patients meeting and those not meeting the full Stevenson criteria [3], we group phenotypes fulfilling the craniofacial part of the Stevenson criteria [3] according to presence or absence of limb malformations. While the focus is on FSS and SHS, patients rediagnosed by review authors with the phenotypically similar conditions distal arthrogryposis types 1A, 1B, and 3 (DA1A, DA1B, and DA3) are included. In this review, no distinction is made between DA1A and DA1B, which are simply termed DA1. DA1 shares the limb malformations of FSS and SHS but lacks craniofacial features; DA3 or Gorden syndrome is associated with cleft palate, as well as similar limb and skeletal malformations as FSS, SHS, and DA1.

## Methods and design

Undertaken as part of the unfunded Freeman-Sheldon syndrome clinical practise guideline development process sponsored by Freeman-Sheldon Research Group, Inc., this systematic review and meta-analysis received institutional review board (IRB) approval from FSRG IRB #1 and was registered with the World Health Orginisation (U1111-1172-4670) and on PROSPERO (CRD42015024740) (Additional file 1: PROSPERO Record Public View), where subsequent amendments are made available. The protocol, including development of actionable clinical questions (Table 1), was prospectively drafted in compliance with Preferred Reporting Items for Systematic Reviews and Meta-Analyses Protocols [7] (Additional file 2: PRISMA-P 2015 Checklist), and all data are reviewed for integrity and consistency by the IRB’s biostatistician. Unless otherwise stated, all actions undertaken are executed by the review authors.

**Table 1:** Actionable guiding clinical questions

1.For patients with suspected or confirmed FSS, SHS, DA1, or DA3, is a craniofacial team, compared with individual speciality referrals, reasonably expected to improve clinical care and to improve achievement of overall treatment outcomes?	
2.For patients with suspected FSS, do plastic surgeons, paediatricians, clinical geneticists, orthopaedic surgeons, anaesthesiologists, or dental surgeons have the highest diagnostic accuracy for FSS and should therefore be the first referral option for providers suspecting a diagnosis of FSS, according to the Stevenson criteria?	
3.For patients with suspected FSS, SHS, DA1, and DA3, are there non-overlapping definable feature (physical findings or historical data) frequency clusters or individual features that are predictive of diagnosis and that providers must be aware of to improve treatment-related decision-making?	
4.For patients with suspected or confirmed FSS, SHS, DA1, or DA3, are there definable feature (physical findings or historical data) frequency clusters or individual features that are predictive of treatment outcome and that providers must be aware of to improve treatment-related decision-making?	
5.For patients with FSS, SHS, DA1, or DA3, is aggressive non-operative therapy (e.g., braces, splints, passive manipulation), compared with surgical correction, reasonably expected to improve achievement of overall treatment outcomes?	
6.For patients with suspected or confirmed FSS, SHS, DA1, or DA3, is neurological consultation, compared with general evaluation, reasonably expected to improve treatment-related decision-making (i.e., distinguishing myopathic processes from primary neurological processes) and outcomes (i.e., monitoring patients with craniosynostosis).	
7.For patients and families affected by FSS, SHS, DA1, or DA3, is early psychiatric consultation, compared with only a general explanation of anticipated clinical course and treatment plans, appropriate to assist in reducing psychosocial sequelae relevant to diagnosis burden?	
8.For preschool and school-age patients with FSS, SHS, DA1, or DA3, is intelligence testing, compared with subjective parent and teacher observation, appropriate to assist in improving access to appropriate academic services?	
9.For patients with FSS, SHS, DA1, or DA3, is ophthalmological consultation, compared with general evaluation, appropriate to assist in improving reconstructive surgery-related decision-making and to improve achievement of overall treatment outcomes?	
10.For patients with FSS, SHS, DA1, or DA3, is otorhinolaryngology consultation, compared with general evaluation, appropriate to assist in improving reconstructive surgery and dysphagia-related decision-making and to improve achievement of overall treatment outcomes?	
11.For patients with FSS, SHS, DA1, or DA3, is paediatric dentistry and oral-maxillofacial surgery referral, compared with general dentistry, required to expect reasonable treatment-related decision-making and reduce dental-related health burdens?	
12.For patients with FSS, SHS, DA1, or DA3, is physiatry referral, compared with orthopaedic surgery evaluation, required to reduce morbidity from extremity and spinal deformities and other functional burdens and to improve achievement of overall treatment outcomes?	
13.For patients with FSS, SHS, DA1, or DA3, is dietectics consultation, compared with general evaluation, appropriate to ensure adequate nutritional intake?	
14.For patients with FSS, SHS, DA1, or DA3, are cardiology and pulmonology consultations, compared with general evaluation, appropriate to reduce consequences of recurrent lower respiratory infections and potential right heart strain?	
15.For patients with FSS, SHS, DA1, and DA3 who have well vascularised equinovarus resistant to non-operative treatment, should referral for fabrication of prosthetic limb without amputation, compared with surgical intervention, be offered to improve achievement of overall treatment outcomes?	
16.Do patients with FSS, SHS, DA1, or DA3, compared with the general population, have special problems that anaesthesia and general emergency medicine providers must consider to expect reasonable treatment-related decision-making and adverse-event free survival?	
17.Do patients with FSS, SHS, DA1, or DA3, compared with the general population, have special imaging findings and considerations that radiologists and pathologists must be aware of that are relevant to improving treatment-related decision-making?	
18.For patients who may have a risk for a FSS, SHS, DA1, or DA3 pregnancy, is genetic counselling, pre-conception molecular testing, post-conception molecular testing, prenatal ultrasound, or elective abortion reasonably expected to improve decision-making and quality of life outcomes?	
19.For delivery of an infant with suspected or confirmed FSS, SHS, DA1, or DA3 or delivery in mother with FSS, SHS, DA1, or DA3, is elective caesarian delivery, compared with vaginal delivery, reasonably expected to reduce foetal and maternal distress and improve adverse-event free survival?	

### Literature search

Material for consideration is identified by searching PubMed and Google Scholar, from December 2014 to July 2015, for all articles in any language relating to FSS, SHS, DA1, and DA3. No advanced search features or limits are used for PubMed because of the paucity of available literature. For Google Scholar, search limits are required because of its broader search inclusion; searches on Google Scholar were limited to articles with search terms appearing in the title. For PubMed (URL: https://goo.gl/FOqJzn) and Google Scholar (URL: http://bit.ly/2eoe1qj), search strategy (Table 2) includes all known syndromic synonyms as search terms, including distal arthrogryposis type 2A, distal arthrogryposis type 2B, distal arthrogryposis multiplex congenita, Freeman-Sheldon syndrome, Freeman-Sheldon, Sheldon-Hall syndrome, whistling face syndrome, craniocarpotarsal dystrophy, craniocarpotarsal dysplasia, cranio-carpo-tarsal dystrophy, cranio-carpo-tarsal dysplasia and cranio-facio-corporal syndrome. Searches are re-run before final analyses, and articles published since the initial search are retrieved for inclusion.

**Table 2:** Search strategy for PubMed database

((((((((“Freeman-Sheldon syndrome”[Supplementary Concept] OR “Freeman-Sheldon syndrome”[All Fields] OR “freeman sheldon syndrome”[All Fields]) OR (“Distal arthrogryposis type 2B”[Supplementary Concept] OR “Distal arthrogryposis type 2B”[All Fields] OR “sheldon hall syndrome”[All Fields])) OR Freeman-Sheldon[All Fields]) OR “distal arthrogryposis multiplex congenita”[All Fields] OR (“Freeman-Sheldon syndrome”[Supplementary Concept] OR "Freeman-Sheldon syndrome”[All Fields] OR “whistling face syndrome”[All Fields])) OR (“Freeman-Sheldon syndrome”[Supplementary Concept] OR “Freeman-Sheldon syndrome”[All Fields] OR “craniocarpotarsal dystrophy”[All Fields])) OR (“Freeman-Sheldon syndrome”[Supplementary Concept] OR “Freeman-Sheldon syndrome”[All Fields] OR “craniocarpotarsal dysplasia”[All Fields])) OR (cranio-carpo-tarsal[All Fields] AND dystrophy[All Fields])) OR (cranio-carpo-tarsal[All Fields] AND dysplasia[All Fields])) OR (cranio-facio-corporal[All Fields] AND “syndrome”[MeSH Terms])	

### Eligibility criteria

Because of the need for detailed patient-level clinical information to meet objectives, articles meeting inclusion criteria included are expected to be limited to observational case reports, negating the utility of evidence grading matrices such as Grading of Recommendations Assessment, Development and Evaluation [8]. No published article is excluded based on design alone, however. Treatment comparison is expected to be limited, as many articles describe neither interventions nor outcomes; those that do cannot be confidently compared, due to high inter-patient and inter-intervention variability. It is not certain that many of the guiding questions will be able to be answered, and the objectives more clearly reflect expectations for what data will be available. There are no restrictions on patient gender, ethnicity, geographical location, religion, socio-economic status, or clinical setting. Most non-English language articles are reviewed by native speakers (Spanish) or translated (Russian, Czech, German) in-house. Asian language articles are unable to be reviewed or translated in-house and are excluded.

### Data extraction

As searches are carried out, citations of unique results are placed in spreadsheets using the current iteration of Google Sheets (Mountain View, CA) for initial independent screening by two review authors to identify those potentially meeting the inclusion criteria. After initial screening, articles retrieved are independently assessed by two review authors for eligibility before inclusion. Discrepancies and disagreements are resolved through discussion, with a third reviewer, or with the ethics director or his designee. Authors of articles being considered are not contacted with data queries. Only published reports of patients, living or deceased, of any age with a stated diagnosis of FSS, SHS, DA1, or DA3 are considered for initial inclusion. Published reports of patients without a stated diagnosis of FSS, SHS, DA1, or DA3 are not considered. Because of previously published reports of diagnostic unreliability for FSS, SHS, DA1, or DA3, only articles with sufficient patient-level clinical information for diagnosis verification are included in the full analysis. Physical findings not evident in the article text are recorded as present, if they are visible in accompanying figures; similarly, if it is clear that a certain physical feature was absent in accompanying figures, it is recorded as not present. Physical findings or historical data present in fewer than five cases are not recorded, and some physical findings or historical data items relatively similar to each other and affecting fewer than five individuals are combined into a single variable. Physical findings or historical data not present or missing for a given variable in a particular patient are both coded as missing. Physical findings are evaluated and described following the Elements of Morphology: Human Malformation Terminology [9–15].

Patient-level clinical information from included studies is extrapolated, based on the standardised Survey of Treatment Outcomes and Practices–Freeman-Sheldon syndrome (STOP-FSS) questionnaire, and entered on a spreadsheet (Google Sheets, Mountain View, CA) for quality assessment and data synthesis. One review author extracts data for each half of the final total of included articles, with another review author verifying data the other review author had entered and coded. The review authors then discuss any differences in their respective appraisals, with discrepancies discussed with a third review author or with the ethics director or his designee.

General types of patient-level clinical information sought (Tables 3 and 4) include published diagnosis, medical speciality of main author, patient congruence with Stevenson criteria [3], demographics, pregnancy complications, birth data, syndromic or potentially syndromic physical findings and their complications, treatment types, anaesthesia types, and overall treatment outcome. Treatments for which data are sought include treatments for any condition or feature actually or potentially complicated by or associated with (primarily or secondarily) FSS, SHS, DA1, or DA3. Such interventions include surgery, physical therapy, and any other organised action to improve health or well-being. Overall treatment outcome is a subjective clinical interpretation by the review authors based on clinical data or opinions presented in the manuscript; patient perception of outcome, if presented in the manuscript, is not considered by the review authors.

**Table 3:** Variables collected on unique papers. Table lists publication-related and review outcome variables tracked for unique papers

Variable	
Year of publication	
Paper code (in-house tracking)	
Article citation (Vancouver style)	
Included/excluded status (in-house tracking)	
Primary published diagnostic term	
Primary published diagnosis	
Reviewer diagnosis	
Molecular diagnosis	
Other diagnosis	
Reason for exclusion	
Number of patients	
Paper type	
Language	
Primary provider (author) speciality	
Country of publication	
Treatment type	
Anaesthesia type	
Overall treatment outcome

**Table 4:** Variables collected for individual patients. Table lists all items for which patient data are sought

General variables	Required features and major distal arthrogryposis variables	Additional clinical variables
Year of publication	Microstomia	Seizures
Paper code (in-house tracking)	Whistling face	Magnetic resonance imaging
Article citation (Vancouver style)	H or V-shaped chin dimple	Computed tomography scan (Give findings, if stated)
Published diagnostic term	Prominent nasolabial folds	Electroencephalography
Primary published diagnosis	Moderately small mouth	Developmental delay
Reviewer diagnosis	Small prominent chin	Mental retardation
Other diagnosis	Neck webbing	Normal intelligence
Reason for exclusion	Ulnar deviation	Reduced anterior-posterior skull distance (radiographically)
Paper type	Camptodactyly	Steep anterior cranial fossa (radiographically)
Language	Hypoplastic or absent flexion creases	Bulging appearance of occiput/deep cerebellar fossa (radiographically)
Primary provider (author) speciality	Overriding fingers at birth	Craniosynostosis
Proband’s country	Overriding toes at birth	Microcephaly
Patient identification (as published, to avoid potential duplication)	Talipes equinovarus	Midline facial nevus
Patient code (in-house tracking)	Talipes calcaneovalgus	Facial asymmetry
Year of birth	Vertical talus	Long face
Age (years)	Metatarsus varus	Triangular face
Karyotype results		Mask-like face
Inheritance status		Flat mid-face
Inheritance pattern		Bulging forehead
Parental consanguinity		Full forehead
Birth order		Superior blepharoptosis
Total number of sibship		Ophthalmoplegia
Maternal age at birth		Blepharophimosis
Paternal age at birth		Downslating palpebral fissures
Gestation (weeks)		Short palpebral fissures
Gestation (if stated as term or non-term)		Epicanthal folds
Mother’s pregnancy illness		Telecanthus
Prenatal polyhydramnios		Ocular hypertelorism
Cardiac abnormalities		Deep set eyes
Caesarean section		Prominent supracilliary ridges
Vaginal delivery		Symetrical subcutaneous elevations of medial frontal areas
Delivery complications		Impaired visual acuity
Breech or transverse presentation		Strabismus
Apgar score (first assessment)		Malar hypoplasia
Apgar score (second assessment)		Hypoplastic alae nasi
Birth weight (kg)		Small nose
Birth weight (if stated as ‘low’ but not given)		Broad nasal root
Birth height (cm)		Broad/depressed nasal bridge
Birth head circumference		Long philtrum
Postnatal growth		Microglossia
Failure-to-thrive		Micrognathia
Most recent weight (kg)		Retrognathia
Most recent weight (if stated as ‘low’ but not given)		Straight mandibular rami
Most recent stature (cm)		Dental crowding
Most recent stature (if stated as ‘low’ but not given)		Malocclusion
Most recent head circumference (cm)		High-vaulted palate
Age at most recent measurements (years)		Narrow palate
		Cleft-lip/palate
		Low set ears
		Posteriorly rotated pinnae
		Attached ear lobules
		Hearing impairment
		Short neck
		Limited cervical range of motion
		Low hairlines
		Kyphosis
		Scoliosis
		Lordosis
		Spina bifida
		Other vertebral anomalies
		Costal abnormalities
		Nipple hypertelorism
		Pectus carinatum
		Pectus excvatum
		Hip dislocation/dysplasia
		Hip contracture
		Leg length/width discrepancy
		Limited knee motion/disloocation
		Patellar anomalies
		Equinovagus (talipes valgus)
		Contracted toes
		Hallux valgus or metatarsus primus Adductus or hallux varus
		Limited shoulder motion/dislocation
		Limited elbow motion/dislocation
		Limited wrist motion
		Cortical thumbs
		Thickened skin on fingers’ flexor surface
		Brachidactyly
		Syndactyly
		Cutaneous syndactyly
		Clinodactyly
		Single palmar crease
		Dysphasia
		Dysphagia
		Gastrointestinal symptoms
		Hernia
		Genitourinary anomalies
		Electromyogram
		Skeletal muscle weakness
		Muscle hypotrophy
		Hypotonia
		Hypertonia
		Upper airway obstruction
		Ear infection and chronic fluid
		Respiratory illness
		Lung disease
		Respiratory distress
		Apnoea
		Early death (state cause)
		*MYBPC1* allelic variation
		*TPM2* allelic variation
		*TNNI2* allelic variation
		*TNNT3* allelic variation
		*MYH3* allelic variation
		Treatment
		Anaesthesia
		Ventilation
		Malignant hyperthermia-triggers used
		Intravenous access
		Clubfoot repair
		Microstomia repair
		Spinal surgery
		Splints, casting, braces, or physiotherapy
		Craniomaxillofacial surgery
		Myringotomies and pressure equalisation tube placement
		Other limb surgery
		General surgery
		Overall treatment outcome

### Risk of Bias (Quality) Assessment

Because of the limitations of case reports, biased risk is not able to be formally assessed, particularly since the quality of case reports and patient data contained therein vary widely. As inter-case variability is significant, case report quality assessment is mostly a clinical decision by one review author and reviewed by a second, based on reviewer knowledge of the syndromes, relative agreement with consensus-based clinical case reporting guideline enough to establish an accurate clinical picture [16], and general clinical experience. In determining speciality of the main author, ambiguity can result, as not all journals report the same type of author information, and certain other judgements, such as overall treatment outcome, are subjective and prone to bias of the original and review authors. Concerns are discussed with a third review author or with the ethics director or his designee.

### Strategy for data synthesis

Most data extracted and generated for this review are nominal, representing binary coding of clinical findings and history to indicate the presence, absence, or non-reporting of each variable sought. Because of high variability of physical features and historical data reported, the small volume of published reports, and anticipated poor diagnostic accuracy in published articles, classic meta-analysis is not possible. Patient-level data are aggregated into a single sample.

Statistical significance for all tests is calculated based on a two-tailed alpha level of 0.05. Chi-squared analysis is used for nominal data, such as physical features or specific aspects of the history. For higher order data, such as measurements and ages, an independent group *t* test or one-way or multiple-way between subject analysis of variance tests is conducted. For each significant *p* value, eta-squared or r-squared is calculated to determine effect size, as appropriate. Pearson correlation, Tukey HSD, and linear regression analysis are also calculated and plotted, as appropriate, to evaluate possible associations between physical findings, specific aspects of the history, treatment type, anaesthesia type, reported and review author diagnosis, and overall treatment outcome. No weighting of variables or models to account for heterogenity are used. Subgroups are each analysed individually in different data files. Data are analysed using the current iterations of PSPP (The Free Software Foundation, Boston, MA) and R: A Language and Environment for Statistical Computing (R Foundation for Statistical Computing, Vienna, Austria) with R Commander [17]. No special or additional commands are used.

### Analysis of subgroups or subsets

Data are analysed by the following diagnosis-related subgroups for both individual cases and papers: published diagnosis, reviewer diagnosis of SHS, reviewer diagnosis of FSS with only craniofacial features, reviewer diagnosis of FSS with craniofacial and upper or lower limb malformations, reviewer diagnosis of FSS with craniofacial and neurological features, and reviewer diagnosis of FSS meeting the full Stevenson criteria [3]. Additionally, the following additional groups are analysed: treatment type, anaesthesia type, treatment outcome, all included cases, all reviewer-diagnosed FSS cases, all retrieved papers, and all included papers fully analysed. Data are also analysed by published diagnosis grouped by major speciality (i.e. paediatrics, medical genetics, plastic surgery, orthopaedics, and anaesthetics) of the main author. Article focus, journal focus and audience, author affiliation, and Google searches of main authors are used by the review authors to make judgements about speciality. This systematic review is undertaken concurrently with an on-going retrospective cohort study using similar data synthesis procedures (NCT01144741).

## Discussion

We expect the need for high quality, detailed patient-level clinical information will limit the effectiveness of this project in assessing the review objectives with any level of reasonable confidence, but we feel this is an acceptable beginning point. Most articles are expected to demonstrate considerable variability in quality of case reports generally, clarity of writing, presence and clarity of accompanying pictorial evidence, details of treatment and outcomes, psychosocial health descriptions, variability of descriptive terminology, and attention to clinical detail and accuracy. All present considerable obstacles, complicating data extraction, coding, and analysis efforts and severely limited significance, power, and utility of the project’s results. The span of years and countries included in the search present additional difficulties of historical context. For example, articles from pre-DNA in the 1930s in England, pre-human genome in the 1980s in Germany, and post-human genome in the 2010s in USA all present patients from differing perspectives and emphasise different aspects of the history, findings, and therapy. Cultural bias, focus of clinical attention, and language each modify text and presentation of articles as well.

We hope this project will begin to enable a more clear outline of knowledge gaps and help prioritise areas for clinical investigation. We feel our methodology may be applicable and helpful for others investigating a condition or phenomena with similar difficulties present in the existing literature that precludes conventional meta-analysis.

## Additional files

Additional file 1: PROSPERO Record Public View. (PDF 130 kb)
Additional file 2: PRISMA-P 2015 Checklist. (PDF 154 kb)

## Abbreviations

DA1: Distal arthrogryposis type 1
DA3: Distal arthrogryposis type 3
FSS: Freeman-Sheldon syndrome
IRB: Institutional review board
SHS: Sheldon-Hall syndrome
