# Supplementary material for: Findings, phenotypes, and outcomes in Freeman-Sheldon and Sheldon-Hall syndromes and distal arthrogryposis types 1 and 3: protocol for systematic review and patient-level data meta-analysis
Source: Syst Rev. 2017 Mar 6;6:46. doi: 10.1186/s13643-017-0444-4 (PMC5339949; doi:10.1186/s13643-017-0444-4)
Supplement: Additional file 1: — PROSPERO Record Public View. (PDF 130 kb) [file 13643_2017_444_MOESM1_ESM.pdf]

PROSPERO International prospective register of systematic reviews

Findings, phenotypes, and outcomes in Freeman-Sheldon and Sheldon-Hall syndromes, and distal arthrogryposis types 1 and 3: protocol for systematic review and patient-level data meta-analysis

Mikaela Poling, José Andrés Morales Corado, Robert Chamberlain

Citation

Mikaela Poling, José Andrés Morales Corado, Robert Chamberlain. Findings, phenotypes, and outcomes in Freeman-Sheldon and Sheldon-Hall syndromes, and distal arthrogryposis types 1 and 3: protocol for systematic review and patient-level data meta-analysis. PROSPERO 2015:CRD42015024740 Available from
http://www.crd.york.ac.uk/PROSPERO_REBRANDING/display_record.asp?ID=CRD42015024740

Review question(s)

For patients with suspected or confirmed FSS, SHS, DA1, or DA3, is a craniofacial team, compared with individual speciality referrals, reasonably expected to improve clinical care and to improve achievement of overall treatment outcomes?

For patients with suspected FSS, do plastic surgeons, paediatricians, clinical geneticists, orthopaedic surgeons, anaesthesiologists, or dental surgeons have the highest diagnostic accuracy for FSS and should therefore be the first referral option for providers suspecting a diagnosis of FSS, according to the Stevenson criteria?

For patients with suspected FSS, SHS, DA1, and DA3, are there non-overlapping definable feature (physical findings or historical data) frequency clusters or individual features that are predictive of diagnosis and that providers must be aware of to improve treatment-related decision-making?

For patients with suspected or confirmed FSS, SHS, DA1, or DA3, are there definable feature (physical findings or historical data) frequency clusters or individual features that are predictive of treatment outcome and that providers must be aware of to improve treatment-related decision-making?

For patients with FSS, SHS, DA1, or DA3, is aggressive non-operative therapy (e.g., braces, splints, passive manipulation), compared with surgical correction, reasonably expected to improve achievement of overall treatment outcomes?

For patients with suspected or confirmed FSS, SHS, DA1, or DA3, is neurological consultation, compared with general evaluation, reasonably expected to improve treatment-related decision-making (i.e., distinguishing myopathic processes from primary neurological processes) and outcomes (i.e., monitoring patients with craniosynostosis).

For patients and families affected by FSS, SHS, DA1, or DA3, is early psychiatric consultation, compared with only a general explanation of anticipated clinical course and treatment plans, appropriate to assist in reducing psychosocial sequllae relevant to diagnosis burden?

For preschool and school-age patients with FSS, SHS, DA1, or DA3, is intelligence testing, compared with subjective parent and teacher observation, appropriate to assist in improving access to appropriate academic services?

For patients with FSS, SHS, DA1, or DA3, is ophthalmological consultation, compared with general evaluation, appropriate to assist in improving reconstructive surgery-related decision-making and to improve achievement of overall treatment outcomes?

For patients with FSS, SHS, DA1, or DA3, is otorhinolaryngology consultation, compared with general evaluation, appropriate to assist in improving reconstructive surgery and dysphagia-related decision-making and to improve achievement of overall treatment outcomes?

For patients with FSS, SHS, DA1, or DA3, is paediatric dentistry and oral-maxillofacial surgery referral, compared with general dentistry, required to expect reasonable treatment-related decision-making and reduce dental-related health burdens?

For patients with FSS, SHS, DA1, or DA3, is physiatry referral, compared with orthopaedic surgery evaluation, required to reduce morbidity from extremity and spinal deformities and other functional burdens and to improve achievement of overall treatment outcomes?

For patients with FSS, SHS, DA1, or DA3, is dietectics consultation, compared with general evaluation, appropriate to ensure adequate nutritional intake?

For patients with FSS, SHS, DA1, or DA3, are cardiology and pulmonology consultations, compared with general evaluation, appropriate to reduce consequences of recurrent lower respiratory infections and potential right heart strain?

For patients with FSS, SHS, DA1, and DA3 who have well vascularised equinovarus resistant to non-operative treatment, should referral for fabrication of prosthetic limb without amputation, compared with surgical intervention, be offered to improve achievement of overall treatment outcomes?

Do patients with FSS, SHS, DA1, or DA3, compared with the general population, have special problems that anaesthesia and general emergency medicine providers must consider to expect reasonable treatment-related decision-making and adverse-event free survival?

Do patients with FSS, SHS, DA1, or DA3, compared with the general population, have special imaging findings and considerations that radiologists and pathologists must be aware of that are relevant to improving treatment-related decision-making?

For patients who may have a risk for a FSS, SHS, DA1, or DA3 pregnancy, is genetic counselling, pre-conception molecular testing, post-conception molecular testing, prenatal ultrasound, or elective abortion reasonably expected to improve decision-making and quality of life outcomes?

For delivery of an infant with suspected or confirmed FSS, SHS, DA1, or DA3 or delivery in mother with FSS, SHS, DA1, or DA3, is elective caesarian delivery, compared with vaginal delivery, reasonably expected to reduce foetal and maternal distress and improve adverse-event free survival?

Searches

Material for consideration has been identified by searching PubMed and Google Scholar, from December 2014 to July 2015, for all articles in any language relating to FSS, SHS, DA1, and DA3.

No advanced search features or limits have been used for PubMed. For Google Scholar, search limits are required because of its broader search inclusion; searches on Google Scholar have been limited to articles with search terms appearing in the title. For PubMed and Google Scholar, the search strategy includes all known syndromic synonyms as search terms, including: distal arthrogryposis type 2A, distal arthrogryposis type 2B, distal arthrogryposis multiplex congenita, Freeman-Sheldon syndrome, Freeman-Sheldon, Sheldon-Hall syndrome, whistling face syndrome, craniocarpotarsal dystrophy, craniocarpotarsal dysplasia, cranio-carpo-tarsal dystrophy, cranio-carpo-tarsal dysplasia, and cranio-facio-corporal syndrome.

Searches have been re-run before final analyses and further articles retrieved for inclusion.

Search results can be viewed at: https://goo.gl/FOqJzn, and http://bit.ly/2eoe1qj

Types of study to be included

Because of the need for detailed patient-level clinical information to meet objectives, articles meeting inclusion criteria included are expected to be limited to observational case reports, negating the utility of evidence grading matrices such as Grading of Recommendations Assessment, Development and Evaluation. No published article is excluded based on design alone, however. Most non-English language articles are reviewed by native speakers

(Spanish) or translated (Russian, Czech, German) in-house. Asian language articles are unable to be reviewed or translated in-house and are excluded.

Condition or domain being studied

First described by Freeman and Sheldon (1938), FSS and SHS are highly variable, rare, and often confused congenital myopathies, despite previous attempts to simplify diagnosis. While Antley et al. (1970) completed a systematic review and statistical analysis of patient-level data from published reports, allelic variation data were not available, and few cases were available for analysis. Stevenson et al. (2006) executed a cohort study of geneticist-referred patients with classic Freeman-Sheldon and Sheldon-Hall syndromes, which limited their cohort. No systematic review has been completed since the genotype-verified clinical diagnostic criteria were published. Unfortunately, few studies involving either or both FSS and SHS exist, and with reported diagnosis unreliable, it would be irresponsible to consider articles describing FSS or SHS, unless diagnoses were independently verified. Thus, a conventional systematic review and meta-analysis methodology was inappropriate in this instance and rigorous patient-level data extraction and aggregation was required.

The systematic review and patient-level meta-analysis objectives are as follows: to (1) determine diagnostic accuracy from 1938-2015, using the Stevenson criteria; (2) define the most common physical findings and their complications amongst patients with FSS; (3) document treatment types and outcomes; and (4) investigate possible frequency clusters for physical findings and their complications. Additionally, a myriad of phenotypes are described as FSS—further complicating literature searches and clinical practice guideline development. To evaluate possible differences with patients meeting and those not meeting the full Stevenson criteria, we group phenotypes fulfilling the craniofacial part of the Stevenson criteria according to presence or absence of limb malformations. While the focus is on FSS and SHS, patients rediagnosed by review authors with the phenotypically similar conditions distal arthrogryposis type 1A, 1B, and 3 (DA1A, DA1B, and DA3) are included. In this review, no distinction is made between DA1A and DA1B, which are simply termed DA1.

Participants/ population

Inclusion: Only published reports of patients, living or deceased, of any age with a stated diagnosis of FSS, SHS, DA1, or DA3 are considered for initial inclusion.

Exclusion: Published reports of patients without a stated diagnosis of FSS, SHS, DA1, or DA3 are not considered. Because of previously published reports of diagnostic unreliability for FSS, SHS, DA1, or DA3, only articles with sufficient IPD for diagnosis verification are included in the full analysis.

Intervention(s), exposure(s)

General types of patient-level clinical information sought includes: published diagnosis, medical speciality of main author, patient congruence with Stevenson criteria, demographics, pregnancy complications, birth data, syndromic or potentially syndromic physical findings and their complications, treatment types, anaesthesia types, and overall treatment outcome. Treatments for which data are sought includes treatments for any condition or feature actually or potentially complicated by or associated with (primarily or secondarily) FSS, SHS, DA1, or DA3. Such interventions include surgery, physical therapy, and any other organised action to improve health or well-being. Overall treatment outcome is a subjective clinical interpretation by the review authors based on clinical data or opinions presented in the manuscript; patient perception of outcome, if presented in the manuscript, is not considered by the review authors.

Comparator(s)/ control

Treatment comparison is expected to be limited, as many articles describe neither interventions nor outcomes; those that do cannot be confidently compared, due to high inter-patient and inter-intervention variability. It is not certain that many of the guiding questions will be able to be answered, and the objectives more clearly reflect expectations for what data will be available.

Context

There are no restrictions on patient gender, ethnicity, geographical location, religion, socioeconomic status, or clinical setting.

Outcome(s)

Primary outcomes
Diagnosis in accordance with the 2006 Stevenson Criteria.

Secondary outcomes
Treatment-related (not just less severe problems due to other factors) clinical or functional improvement.

Data extraction, (selection and coding)
As searches are carried out, citations of unique results are placed in spreadsheets using the current iteration of Google Sheets (Mountain View, CA) for initial independent screening by both review authors to identify those potentially meeting the inclusion criteria. After initial screening, articles retrieved are independently assessed by both review authors for eligibility before inclusion. Discrepancies and disagreements are resolved through discussion or with a third reviewer or with the ethics director or his designee. Authors of articles being considered are not contacted with data queries. Only published reports of patients, living or deceased, of any age with a stated diagnosis of FSS, SHS, DA1, or DA3 are considered for initial inclusion. Published reports of patients without a stated diagnosis of FSS, SHS, DA1, or DA3 are not considered. Because of previously published reports of diagnostic unreliability for FSS, SHS, DA1, or DA3, only articles with sufficient patient-level clinical information for diagnosis verification are included in the full analysis. Physical findings not evident in the article text are recorded as present, if they are visible in accompanying figures; similarly, if it is clear that a certain physical feature was absent in accompanying figures, it is recorded as not present. Physical findings or historical data present in fewer than five cases are not recorded, and some physical findings or historical data items relatively similar to each-other and affecting fewer than five individuals are combined into a single variable. Physical findings or historical data not present or missing for a given variable in a particular patient are both coded as missing. Physical findings are evaluated and described following Elements of Morphology: Human Malformation Terminology.

Patient-level clinical information from included studies is extrapolated, based on the standardised Survey of Treatment Outcomes and Practices – Freeman-Sheldon syndrome (STOP-FSS) questionnaire, and entered on a spreadsheet (Google Sheets, Mountain View, CA) for quality assessment and data synthesis. One review author extracts data for each half of the final total of included articles, with the other review author verifies data the other review author had entered and coded. Both review authors then discuss any differences in their respective appraisals. Discrepancies are discussed with a third or fourth reviewer or with the ethics director or his designee.

Risk of bias (quality) assessment
Because of the limitations of case reports, bias risk is not able to be formally assessed, particularly since the quality of case reports and patient data contained therein vary widely. As inter-case variability is significant, case report quality assessment is mostly a clinical decision by one review author and reviewed by the second, based on reviewer knowledge of the syndromes, relative agreement with Consensus-based Clinical Case Reporting Guideline enough to establish an accurate clinical picture, and general clinical experience. In determining speciality of the main author, ambiguity can result, as not all journals report the same type of author information, and certain other judgements, such as overall treatment outcome, are subjective and prone to bias of the original and review authors. Concerns are discussed with a third author or with the ethics director or his designee.

Strategy for data synthesis
Most data extracted and generated for this review are nominal, representing binary coding of clinical findings and history to indicate the presence, absence, or non-reporting of each variable sought. Because of high variability of physical features and historical data reported, the small volume of published reports, and anticipated poor diagnostic accuracy in published articles, classic meta-analysis is not possible. Patient-level data is aggregated into a single sample.

Statistical significance for all tests is calculated based on a two-tailed alpha level of 0.05. Chi-squared analysis is used for nominal data, such as physical features or specific aspects of the history. For higher order data, such as measurements and ages, an independent groups t-test or one-way or multiple-way between subjects analysis of variance tests is conducted. For each significant p-value, eta-squared or r-squared is calculated to determine effect size, as appropriate. Pearson correlation, Tukey HSD, and linear regression analysis is also calculated and plotted, as appropriate, to evaluate possible associations between physical findings, specific aspects of the history, treatment type, anaesthesia type, reported and review author diagnosis, and overall treatment outcome. No weighting of

variables or models to account for heterogenity are used. Subgroups are each analysed individually in different data files. Data are analysed using the current iterations of PSPP (The Free Software Foundation, Boston, MA) and R: A Language and Environment for Statistical Computing (R Foundation for Statistical Computing, Vienna, Austria) with R Commander [No special or additional commands are used].

Analysis of subgroups or subsets

Data are analysed by the following diagnosis-related subgroups for both individual cases and papers: published diagnosis, reviewer diagnosis of SHS, reviewer diagnosis of FSS with only craniofacial features, reviewer diagnosis of FSS with craniofacial and upper or lower limb malformations, reviewer diagnosis of FSS with craniofacial and neurological features, and reviewer diagnosis of FSS meeting the full Stevenson criteria. Additionally, the following additional groups are analysed: treatment type, anaesthesia type, treatment outcome, all included cases, all reviewer diagnosed FSS cases, all retrieved papers, and all included papers fully analysed. Data are also analysed by published diagnosis grouped by major speciality (i.e., paediatrics, medical genetics, plastic surgery, orthopaedics, and anaesthesia) of the main author. Article focus, journal focus and audience, author affiliation, and Google searches of main authors are used by the review authors to make judgements about speciality. This systematic review is undertaken concurrently with an on-going retrospective cohort study using similar data synthesis procedures (NCT01144741).

Dissemination plans

A manuscript version of the final protocol will be submitted to an appropriate peer-reviewed medical journal on research methodologies. Upon completion of the systematic review, manuscripts describing the results and their implications will be submitted to appropriate peer-reviewed journals in craniofacial surgery and rare diseases. Any resulting published manuscripts (or a summary thereof) and supplemental data will also be made available on the review authors' ResearchGate.net profile pages.

Contact details for further information

Mikaela Poling

6670 Old Elkins Road

Buckhannon, WV 26201

USA

poling_mi@fsrgroup.org

Organisational affiliation of the review

Freeman-Sheldon Research Group, Inc.

http://www.fsrgroup.org/

Review team

Miss Mikaela Poling, FSRG deGruyter-McKusick Institute of Health Sciences
Dr José Andrés Morales Corado, FSRG deGruyter-McKusick Institute of Health Sciences
Dr Robert Chamberlain, FSRG deGruyter-McKusick Institute of Health Sciences

Anticipated or actual start date

19 December 2014

Anticipated completion date

20 January 2017

Funding sources/sponsors

Undertaken as part of the unfunded Freeman-Sheldon syndrome clinical practice guideline development process and sponsored by Freeman-Sheldon Research Group, Inc., systematic review and meta-analysis received institutional

review board (IRB) approval from FSRG IRB #1. All data are reviewed for integrity and consistency by the IRB's biostatistician. Unless otherwise stated, all actions undertaken are executed by the review authors.

Conflicts of interest
None known

Other registration details
WHO Universal Trial Identifier Number (UTN): U1111-1172-4670

Language
English, Russian, Spanish

Country
United States of America

Subject index terms status
Subject indexing assigned by CRD

Subject index terms
Arthrogryposis; Humans

Any other information
The authors have undertaken the project to aid in developing clinical practice guidelines, as part of their academic requirements for advanced study in clinical and applied physiology.

Stage of review
Ongoing

Date of registration in PROSPERO
22 July 2015

Date of publication of this revision
11 January 2017

DOI
10.15124/CRD42015024740

Stage of review at time of this submission	Started	Completed
Preliminary searches	Yes	Yes
Piloting of the study selection process	Yes	Yes
Formal screening of search results against eligibility criteria	Yes	Yes
Data extraction	No	No
Risk of bias (quality) assessment	No	No
Data analysis	No	No

PROSPERO
International prospective register of systematic reviews
The information in this record has been provided by the named contact for this review. CRD has accepted this information in good faith and registered the review in PROSPERO. CRD bears no responsibility or liability for the content of this registration record, any associated files or external websites.
